# Recent Updates on Treatment of Ocular Microbial Infections by Stem Cell Therapy: A Review

**DOI:** 10.3390/ijms19020558

**Published:** 2018-02-13

**Authors:** Seoh Wei Teh, Pooi Ling Mok, Munirah Abd Rashid, Mae-Lynn Catherine Bastion, Normala Ibrahim, Akon Higuchi, Kadarkarai Murugan, Rajan Mariappan, Suresh Kumar Subbiah

**Affiliations:** 1 Department of Biomedical Science, Universiti Putra Malaysia, 43400 UPM Serdang, Selangor, Malaysia; seohwei1208@gmail.com; 2 Genetics and Regenerative Medicine Research Centre, Universiti Putra Malaysia, 43400 UPM Serdang, Selangor, Malaysia; 3 Department of Clinical Laboratory Sciences, College of Applied Medical Sciences, Aljouf University, 72442 Sakaka, Aljouf Province, Saudi Arabia; 4 Department of Ophthalmology, Faculty of Medicine, UKM Medical Center, 56000 Cheras, Kuala Lumpur, Malaysia; munirah12@gmail.com (M.A.R.); maelynnbdr@gmail.com (M.-L.C.B.); 5 Department of Psychiatry, Universiti Putra Malaysia, 43400 UPM Serdang, Selangor, Malaysia; normala_ib@upm.edu.my; 6 Department of Chemical and Materials Engineering, National Central University, No. 300, Jhongda RD., Jhongli, 32001 Taoyuan, Taiwan; akon.higuchi@gmail.com; 7 Department of Zoology, Thiruvalluvar University, Serkkadu, 632 115 Vellore, India; kmvvkg@gmail.com; 8 Biomaterials in Medicinal Chemistry Laboratory, Department of Natural Products Chemistry, School of Chemistry, Madurai Kamaraj University, Madurai, 625 021 Tamil Nadu, India; rajanm153@gmail.com; 9 Department of Medical Microbiology and Parasitology, Universiti Putra Malaysia, 43400 UPM Serdang, Selangor, Malaysia

**Keywords:** ocular microbial infections, endophthalmitis, stem cells, inflammation, tissue regeneration

## Abstract

Ocular microbial infection has emerged as a major public health crisis during the past two decades. A variety of causative agents can cause ocular microbial infections; which are characterized by persistent and destructive inflammation of the ocular tissue; progressive visual disturbance; and may result in loss of visual function in patients if early and effective treatments are not received. The conventional therapeutic approaches to treat vision impairment and blindness resulting from microbial infections involve antimicrobial therapy to eliminate the offending pathogens or in severe cases; by surgical methods and retinal prosthesis replacing of the infected area. In cases where there is concurrent inflammation, once infection is controlled, anti-inflammatory agents are indicated to reduce ocular damage from inflammation which ensues. Despite advances in medical research; progress in the control of ocular microbial infections remains slow. The varying level of ocular tissue recovery in individuals and the incomplete visual functional restoration indicate the chief limitations of current strategies. The development of a more extensive therapy is needed to help in healing to regain vision in patients. Stem cells are multipotent stromal cells that can give rise to a vast variety of cell types following proper differentiation protocol. Stem cell therapy shows promise in reducing inflammation and repairing tissue damage on the eye caused by microbial infections by its ability to modulate immune response and promote tissue regeneration. This article reviews a selected list of common infectious agents affecting the eye; which include fungi; viruses; parasites and bacteria with the aim of discussing the current antimicrobial treatments and the associated therapeutic challenges. We also provide recent updates of the advances in stem cells studies on sepsis therapy as a suggestion of optimum treatment regime for ocular microbial infections.

## 1. Introduction

Ocular microbial infections can cause endophthalmitis (an inflammation of the interior of the eye), an inflammatory reaction that will lead to visual disturbance and potentially produce blinding outcome [1]. Inner eye inflammation can damage the ocular layers which are important for visual processing, such as cornea and retina, which is irremediable by common antimicrobial treatment [2]. Thus, a potent management regime is urgently required, which could be discovered in stem cells treatment.

The eye is one of the major sensory organs in the human body responsible for visual functions, which has a spherical structure. The cornea is the outmost transparent layer of the eyes that refract light onto the retina [3,4]. The retina is the inner coat of the ocular tunics, comprised of 10 different layers of highly organized and complex neurons interconnected by synapses, with the innermost layer of light-sensitive rods and cones photoreceptor cells. Rods support the perception of black-and-white image while cones are responsible for color vision. Neural signals produced are then processed by other retinal neurons in the visual pathway [3,4]. Ocular microbial infections can lead to opacification and intraocular tissue damages which in turn affect retinal encoding and light processing and eventually produces irreversible vision loss [1,5]. Considering the structure and the elements of the retina, conditions spread to the retina, particularly disintegrating the architecture of the retina represent the most tragic clinical manifestations among the intraocular infections [1]. Although the rate of mortality caused by microbial infections in the eyes is relative low, the resulted visual loss intensely affects the quality of life (QOL) of the patients [5]. Hence, effective therapeutic strategies should be sought in alternative remedies such as stem cells.

Introduction of infectious pathogens to the eyes either exogenously (post-traumatic or post-operative), or endogenously (hematogenous microbial dissemination from a distant infected body part) causes chronic inflammation of the eyes [6,7]. The severe and lasting inflammatory response in the eyes is a potentially devastating condition as it may result in edema, opacity and eventually ocular tissue damages [1,5]. Consequently, the inflammation caused by microbial infections intensely aggravate the quality of eye vision of an affected individual. The inflammatory response could lead to rapid loss of visual acuity within several days [8,9] and could even result in retinal detachment within 12 h [10], depending on the severity of the infection. Therefore, prompt and effective treatment should be given to the patient after an infection.

**Figure 1:**
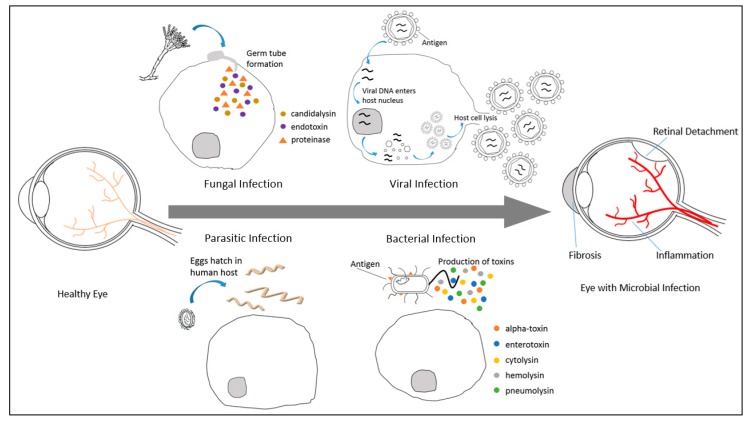
Molecular pathogenesis of ocular microbial infections. The infectious microorganisms can cause inflammation, retinal detachment and tissue fibrosis in affected eyes. Fungus attacks host cells by the formation of germ tube to penetrate and release endotoxins and proteinases to the cells [12,13]. Whereas, virus attachment to host cells membrane facilitates viral DNA integration to the host nucleus, virus reproduction in host and subsequently host cells lysis to release the produced progeny [14]. On the other hand, the eggs of certain parasites can hatch in human host and trigger severe inflammatory reaction and tissue necrosis in the host [15]. Finally, the presence of antigen on bacterial membrane and the production of toxins can cause inflammation and induce damage in the ocular tissue [16].

The infectious microorganisms cause intensive tissue inflammation, structural disturbance and ocular tissue remodeling by the stimulation of tissue fibrosis [11]. Upon invasion into the host eyes, secretion of fungal endotoxins and proteinases can trigger the release of interleukin (IL)-1α, IL-1β and IL-17 in the eye [12,13], resulting in intense inflammation (Figure 1). Whereas, viral capsid proteins can attach and penetrate host cells to integrate viral DNA into the host nucleus, after which the host cells will undergo lysis to release the produced progeny [14]. In addition, certain parasites also induce expression of IL-12 and tumor necrosis factor-α (TNF-α) during infection, causing tissue necrosis [15]. On the other hand, bacteria can excrete toxins and antigenic proteins capable of stimulating inflammatory reactions and suffice to induce damage in the ocular tissue [16].

When human eyes are infected by microorganisms, the injured tissue undergo healing by the release of cytokines, chemokines and growth factors [17]. Infectious microorganisms and infected cells are removed by neutrophils and monocytes via macrophage differentiation [18]. Macrophage differentiation activates fibrogenesis and angiogenesis, induces re-epithelialization and the secretion of connective tissue proteins such as vimentin and collagens I and III [19]. Fibrotic response and tissue scarring due to excessive extracellular matrix deposition results in opacity in the patient eyes [11]. Hence, extrinsic medication is required to promptly overcome the infections, halt progression of tissue damage by microbes and reduce scarring. 

Infectious pathogens could be killed by antimicrobials in which local therapy can be administered via ocular injections, oral or intravenous medications. Treatment for endogenous intraocular infections, meanwhile, can be provided at the primary site of infection [7,20,21]. Despite successful elimination of most microbes, damages to the ocular layers can never be reverted [22,23]. In adult mammals, the neuroretina and retinal pigment epithelium (RPE) do not support neurogenesis as observed in the lower vertebrates [24]. Microbial infections affecting the retina can cause permanent visual impairment when the photoreceptor cells do not spontaneously regenerate after experiencing the unalterable damage [24]. Furthermore, in some serious cases of microbial infections such as Histoplasmosis, laser treatment is required. Even with the repeated laser therapy, the procedure is inadequate to heal the ocular tissue damage [2]. In other cases, retinal prosthesis is needed to replace the infected area following the removal of the damaged region surgically [25]. However, this invasive strategy has the possible drawback of imposing heat damage to the retinal tissue due to close proximity of the implant to retina layers within the compact ocular space [25].

Owing to the limitations possessed by the conventional antimicrobial and surgical approaches, the battle against ocular infections due to contaminating microorganisms is ought to be participated by stem cell therapy, helpful in the successful management of microbial diseases in many recent studies [26,27,28,29]. The idea of potent therapeutic arsenal by stem cells is also supported by their self-renewal and regenerative potential [30,31,32]. Thus, the concept to suppress inflammation and replace the infection damaged photoreceptor cells and RPE by stem cells transplantation represents a highly appealing therapeutic intervention. This review emphasizes the urgent need of an alternative strategy in stem cells treatment to supplement the conventional antimicrobial management, in treating ocular microbial infections. 

## 2. Challenges of Conventional Antimicrobial Treatments for Ocular Microbial Infections

Ocular microbial infections are caused by a variety of pathogenic microorganisms such as fungi [6,7,33], viruses [20,34,35], parasites [36,37,38] and bacteria [39,40,41]. These microbes reach the inner eyes following intraocular surgery [41,42,43,44,45,46,47,48], trauma [49,50], or access by the metastatic spread from other affected anatomical regions [39,51,52,53,54,55,56] and give rise to different effects in patients according to the virulence of microorganisms and the patient immune status [22,23,57,58,59,60]. The primary symptoms of these infections damaging the inner eyes is blurred vision and rapidly deteriorating visual acuity within a few days of infections [8,9]. During the onset of the infections, immune cells and other immunologically active substances infiltrate into the intraocular layers [61] and result in inflammatory reactions. Inflammation-mediated ocular opacification hinders the clear image formation on the retina for a meaningful visual perception [61]. Moreover, retinal tissue damage involving the photoreceptor cells and RPE induced by inflammatory response impedes the basic light-processing photochemical pathway of vision [61]. The outcomes of these complications are the irreversible loss of vision in the affected individuals. 

Intraocular infections caused by microorganisms are usually treated by antimicrobials, which produce variable yet poor results in patients due to several challenges encountered during the course of treatment [21,62]. The challenges of conventional antimicrobial therapy lie on the fact that even with aggressive therapy, damaged tissue could not be recovered and frequently results in vision impairment [2,22,23,63,64]. Depending on the severity of the infections, antimicrobials treatment could take a long period of time to effectively eradicate the pathogenic agents in the eyes [7,21,62,65,66,67]. For some infections, visual disturbance will recur despite laser procedures or surgery treatment [2,64]. There are many hypothetical questions about ocular infections in the scientific community, one of the major questions has to be “is conventional antimicrobials therapy enough for the treatment of ocular tissue damage?” The outcomes of treatment vary due to the age of the patient, species of pathogens, duration between injury and treatment and the extent of the ocular tissue damage [68]. Delay in delivery of efficient therapeutic management could lead to poor and potentially blinding outcome [6,43,54,69,70,71]. 

The offending pathogens affecting the eyes are conventionally combated with antimicrobial agents, which demonstrated low efficiency due to problems in drug administration and diffusion to infected site [72,73,74]. At the onset of microbial infections in the eyes where the causative microbes have not been identified, the antimicrobial drug administered is decided empirically. However, difficulty in correlating infection clinical manifestations and culture results provides minimal assistance on antimicrobial decision [75]. In most of the cases, visual impairment remains as the common outcome even when broad-spectrum antimicrobials were used [45,76]. Furthermore, severe inflammatory reaction occurs in the inner eyes leads to edema and exacerbates the ocular condition [5]. Therefore, anti-inflammatory drugs are often administered concurrently with a high dose of antimicrobials to suppress the intraocular inflammation while killing the offending agents. Nevertheless, clinical evidences have proven that these drugs do not pose any consequences on the inflammation-derived enzymes and toxins that adversely influence the retinal architecture and function [16,77]. Destructed retinal structure and neuroretinal function inevitably lead to the result of blindness.

Despite enormous effort in the science and medicine to heal ocular microbial infections, the severity of ocular diseases continues to pose various risks and complications to the infected individuals. This is due to the delicate ocular cells such as photoreceptor cells and RPE, which possess extreme sensitivity towards the insulting microorganisms, the inflammatory response elicited there upon and the high doses of antimicrobials administered onsite [45,78,79,80]. The traditional treatments of endophthalmitis includes intravitreal administration of antimicrobial agents [45,81,82,83] and simultaneous systemic drug injection [6,44]. However, the isolation of the retina by an avascular vitreous and anterior chamber hamper the effective penetration of the potentially effective antimicrobials to the infected site [72,73,74] following drug injection systemically. Such unique feature as blood-ocular fluid barrier represents a major obstruction for the antimicrobial agents to be delivered by the systemic circulation to the blood-rich retinal layers. The inflammation in the inner eye enhances blood-ocular fluid barrier permeability, thereby promoting the antimicrobial diffusion into the vitreous cavity [74]. However, the intravitreal levels of antimicrobial following direct injection of drugs into the systemic circulation are highly variable and often failed to achieve the minimal inhibitory concentration for various infectious microbes [46]. The physiological challenges of and complications resulted from antimicrobial drugs administrated locally and intravenously exert significant effects on the extension of treatment duration. This, in turn, gives rise to adverse drug reactions, including drug toxicity and drug susceptibility. The most blatant examples of drug toxicity are demonstrated by amphotericin B usage in *Cryptococcus neoformans* infections [84,85] and the utilization of foscarnet and cidofovir against cytomegalovirus (CMV) [57,86]. All the challenges possess by the anatomical structure of the human eye and drug delivery serve as immense hurdles on the traditional antimicrobials therapy to heal endophthalmitis. The invention of a new modality to fight against ocular microbial infection in stem cell therapy is, thus, in pressing need.

### 2.1. Ocular Fungal Infections and the Challenges of Conventional Antifungal Treatment 

Human eyes are vulnerable to microbial attack and fungus represents one of the most frequent causative agents among the microorganisms infecting the delicate ocular tissues [33]. Fungal infections in the eyes are commonly treated with antifungal, however, the effective treatments are not successfully delivered due to various challenges. The common pathogenic fungus causing severe infections are *Candida* sp. [7], *Aspergillus* sp. [33], *Cryptococcus* sp. [84] and *Histoplasma* sp. [2] (Table 1). Among all, the most widely seen fungus species causing endophthalmitis is *Candida* sp. such *as Candida albicans*, which appear as dermal commensal microbes in healthy individuals and opportunistic pathogens in immune-deficient patients [60]. Fungal infections in the eyes may be caused by hematogenous spread from a distant body area harboring infection caused by *Candida* sp. or *Aspergillus* sp. and produce ocular manifestations such as white infiltrates in the inner ocular cavity and hemorrhages [6,7,33]. 

Upon *Candida* sp. adhesion to host epithelial cell walls, germ tubes are formed, candidalysin, endotoxins and proteinases are secreted [12,13,100]. During infection, up-regulation of IL-1α, IL-1β, IL-17 and TNF can cause ocular tissue destruction [12,13]. Ocular candidiasis can be overcome by antifungal caspofungin, micafungin or anidulafungin [7]. On the other hand, antifungal voriconazole or posaconazole is used against *Aspergillus* sp. [7,33,87,88], administered either intravenously or orally. These antifungal treatments require prescription over a long period of time that spans across few months [7], therefore, a more effective intervention should be sought in stem cells for more rapidly healing mechanisms in the affected patients.

*Cryptococcus neoformans* infecting the eyes can be eliminated by intravenous amphotericin B. However, it demonstrates poor diffusion into the vitreous cavity, toxic to human and can cause complications such as renal failure and anaphylaxis in patients receiving high dosage or exposed to long-term therapy [101,102,103]. On the other hand, the use of flucytosine as alternative treatment for *Cryptococcal* infections has been reported to be associated with rapid development of antifungal resistance [84,85]. Even with the drawbacks of these antifungal therapy, many clinicians are still using them to treat infections. Stem cell therapy should be looked into for its effectiveness in the elimination of pathogens.

*Histoplasma* *capsulatum* infections, commonly occurring in patients with compromised immune system, represent the most critical ocular fungal infection. Patients commonly show symptoms of chronic inflammation, hemorrhage and rapid visual impairment [2]. An acquired immune deficiency syndrome (AIDS) patient was reported to have developed retinitis from the disseminated pulmonary *Histoplasma capsulatum* and CMV infection and demonstrated characteristic of creamy white infiltrates with histoplasma yeast cells, lymphocytes and histiocytes in retinal layers. The patient died within a month from the opportunistic infection [64]. In cases of ocular histoplasmosis, the adopted management is usually repetitive laser cauterization of the affected area to slow the macula destruction process [2]. Despite the laser procedures, the repair of the induced damage is still unfeasible. The severity of ocular fungal infections and the limitations of traditional therapeutic intervention call for the discovery of a more potent treatment approach in stem cell therapy for the substantial recovery of ocular tissue damaged by insulting microorganisms.

### 2.2. Ocular Viral Infections and the Challenges of Conventional Antiviral Treatment 

CMV retinitis caused by CMV is usually seen in hosts with compromised immune systems [62]. Frequent ocular manifestations include diffusion of white granular lesion over 8 months, vessel sheating and hemorrhages. A case report stated that within an average of 10 weeks, retinal scar was produced in two patients with a reduction in visual acuity in 50% of the eyes [9]. CMV retinitis progressively results in full-thickness retinal necrosis followed by retinal vascular endothelial cells loss and ultimately retinal detachment in the late stage [9,59,104]. CMV first targets on retinal vascular endothelial cells and spread through retinal vasculature to the RPE in the development of retinal vasculopathy and CMV retinitis [104]. Initially, FasL-mediated apoptosis of RPE could protects host against immune invasion stimulated by CMV. However, this mechanism fails to completely clear CMV in RPE and elicit further immune responses which leads to retinitis [105]. High secretion of TNF-α and interferon-γ (IFN-γ) in immunocompromised patients could aggravate the condition by increasing the sensitivity of RPE to FasL pathway, causing retinal necrosis [14].

Ganciclovir [21], foscarnet [57], cidofovir [62] and fomivirsen [89] serve as the common management options to combat CMV endophthalmitis. Intravenous or intravitreous administration of ganciclovir takes more than 3 weeks to completely eliminate the pathogenic agents [21]. Whereas, intravenous delivery of foscarnet causes nephrotoxicity and electrolyte disturbance [57]. The side effects of nephrotoxicity and the outcome of sight-threatening uveitis and hypotony are also observed with cidofovir treatment [62]. Furthermore, drug resistance can develop specifically in patients with impaired immune function. When AIDS patient is infected with CMV endophthalmitis, highly active antiretroviral therapy (HAART) should be initiated immediately. Nevertheless, HAART is highly associated to the development of immune recovery uveitis [22,23,63] and eventually results in blindness. Currently, patients infected with virus are still treated with these antiviral drugs although there are reports of complications. Stem cell therapy should be sought as a more effective therapeutic regime for ocular infections.

**Figure 2:**
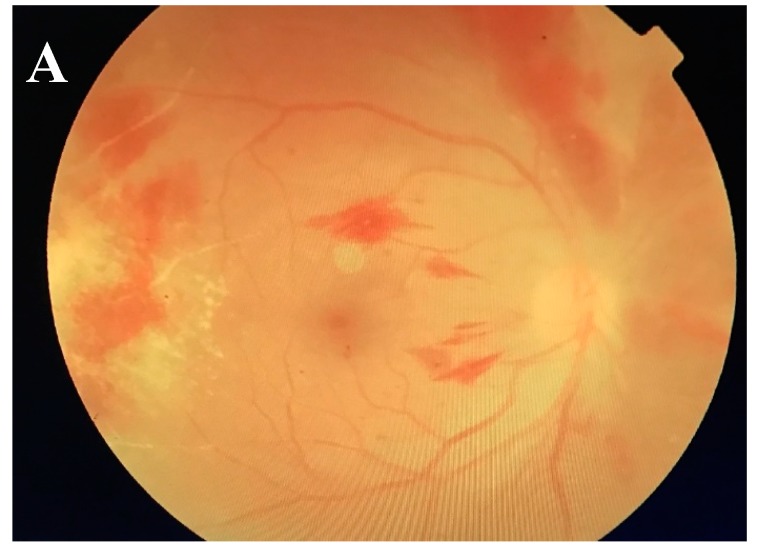

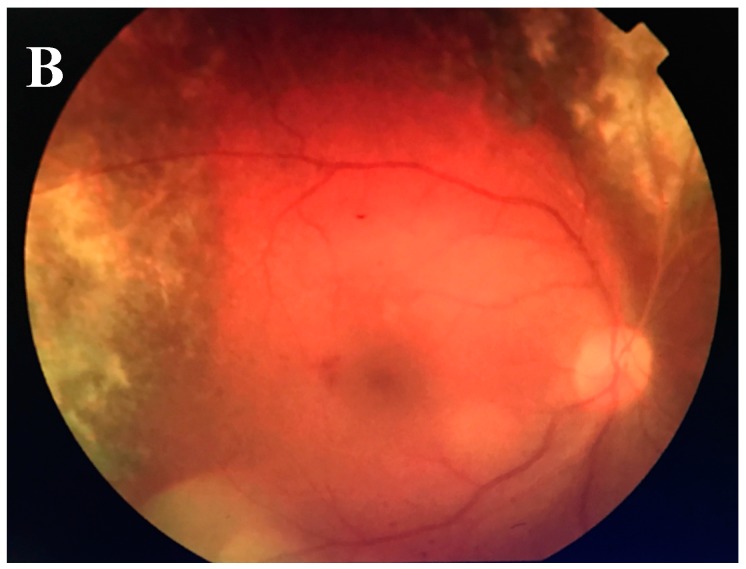
Fundus of patient with CMV retinitis. (**A**) Before antimicrobial treatment, the patient had vitritis grade 1, retinitis in the temporal periphery, vasculitis, retinal hemorrhages and optic disc swelling. (**B**) After antimicrobial treatment, the fundus shows retinal scarring.

A 41-year old man from Sabah, Malaysia, with history of disseminated *Cryptococcal meningitis* and *Klebsiella septicaemia*, was infected with CMV retinitis and treated in Universiti Kebangsaan Malaysia Medical Center (UKMMC). The patient presented floaters in his right eye for 1 month, with vision of 6/18 and pin hole of 6/9 N6 for right eye. Whereas, his left eye had vision of 6/24 and pinhole 6/18 N6. Examination of the HIV positive patient’s eyes revealed fine white keratic precipitate and anterior chamber cells bilaterally. On fundus examination, there was vitritis grade 1, retinitis, vasculitis, retinal hemorrhages and optic disc swelling (Figure 2A). Intravitreal tap also showed positive result for CMV analysis. The patient was treated with intravitreal ganciclovir (0.1 mL/20 mg) and oral valganciclovir (900 mg BD) for 6 weeks. Simultaneously, HAART was administered to increase cluster of differentiation (CD)4 and CD8 counts. On day 18 of the treatment, the right eye of the patient developed superotemporal retinal detachment from atrophic hole and underwent scleral buckle procedure for repairing. However, the right eye retina developed redetachment and the patient was then subjected to laser photocoagulation and gas tamponade. Upon completion of 6 weeks oral valganciclovir treatment, the retina demonstrated scarring (Figure 2B), with vision 6/12 pinhole 6/9 for right eye and vision 6/9 pinhole 6/9 for left eye. This case study has proven that the traditional antiviral therapy is not very effective and failed to completely repair damages even after a long duration of treatment. Therefore, stem cell therapy may be adopted in treating ocular infections to complement the current therapeutic management.

In addition to CMV infection, herpetic and varicella viral infections are likewise dreadful and can result from systemic infection regardless of the immune status of the host. Herpes simplex virus (HSV), herpes zoster virus (HZV) and varicella zoster virus (VZV) can lead to acute retinal necrosis (ARN) in immune-competent individuals and progressive outer retinal necrosis (PORN) in patients with compromised cell-mediated immunity [20,34,35,55,62,106,107]. The rapidly progressive retinitis was featured by retina tissue sparing, retinal vasculature, hemorrhage, massive necrosis and the complication of rhegmatogenous retinal detachment [107]. 

Patients are commonly given oral valaciclovir [62,90], famciclovir [62,91], or intravenous acyclovir therapy [62,107], which requires 7 to 12 weeks of treatment period [67]. These strategies have been shown to produce poor outcomes [107,108,109], mainly due to drug resistance [110]. Alternatively, intravitreal foscarnet [20] is employed to combat the infections. Laser treatment and surgery may also be required to repair rhegmatogenous retinal detachments. Prophylactic argon laser could be used to minimize the risk of retinal detachment but its use is controversial [8,111,112,113,114]. Meanwhile, cryosurgery procedure will simultaneously destroy the functioning retina [115]. The prolonged period of antiviral therapy and ineffective operative strategy validate the need of stem cells intervention as a useful regime in treating microbial infections.

### 2.3. Ocular Parasitic Infections and the Challenges of Conventional Antiparasitic Treatment 

Microbial infections in the inner eyes could be caused by various parasites, which produce very serious ocular manifestations within a short period of time. The most common species of parasite causing endophthalmitis are *Toxocara canis* [37,38], *Toxocara cati* [92,116] and *Toxoplasma gondii* [62,117]. Toxocariasis caused by *Toxocara canis* (roundworm from dogs) and *Toxocara cati* (roundworm from cats) can cause uveitis, tissue scarring and loss of vision within 2 days [116]. Prompt and useful treatment is required to prevent the rapid visual impairment induced by the parasite. The infections are counter-attacked by albendazole or thiabendazole with corticosteroid anti-inflammatory agents applied topically or periocularly [37,38,92]. Direct laser photocoagulation is adopted in the cases in which mobile larvae are seen [36]. 

Whereas, *Toxoplasma gondii* induced ocular toxoplasmosis is mainly observed in immune-compromised hosts, causing hemorrhage, scarring and tissue destruction in retinitis [62]. Toxoplasmosis can also be acquired during pregnancy, leading to congenital infection in the newborn. Macula involvement is widely seen, where the developing fetus will experience devastated central vision [117]. At the early stage of parasitic infection, apoptosis mechanisms and Fas/FasL pathways serve as host protective mechanism. However, the overexpression of Fas and FasL in response to *Toxoplasma gondii* infections could result in excessive ocular tissue damage [118,119]. Moreover, host monocytes phagocytosis of toxoplasma tachyzoites stimulated the production of IL-12 and TNF-α [15]. The infectious diseases are commonly treated with the combination of sulfadiazine with pyrimethamine, sulfamethoxazole with trimethoprim or azithromycin with pyrimethamine [93,94,95]. However, clinical trials have provided inadequate evidences where the medications can improve the outcome of the infections [77]. Over 80% of patients experience relapses for more than 5 years [117]. Moreover, the killing of parasites could trigger an intensified inflammatory response in the eyes, thus render the treatment strategies debatable. The clinical data has proven that antimicrobials are insufficient in overcoming infections due to parasites. To reduce inflammation and prevent ocular tissue damage while eliminating the parasites in the inner ocular layers, stem cell therapy should be considered as an ideal treatment for parasitic endophthalmitis.

### 2.4. Ocular Bacterial Infections and the Challenges of Conventional Antibiotics Treatment 

Bacteria such as *Enterococci* [120], *Staphylococci* [44,47,96] and *Bacilli* [68,121] are common cause of infectious diseases in the eyes. Among all, *Staphylococcus aureus*, *Bacillus cereus* and gram-negative bacteria, such as *Escherichia coli*, *Neisseria meningitides* and *Klebsiella* species are responsible for *endogenous* retinal infections spread from other anatomical area [6,39,40,51,56]. For instance, drug abusers may contract *Bacillus* infection from contaminated drug taken intravenously or from injection paraphernalia [52]. The alpha-toxin of *Staphylococcus aureus* [16], cytolysin of *Enterococcus faecalis* [122] and pneumolysin of *Streptococcus pneumoniae* [123] secreted during infection can induce intensive injury to ocular tissue. The production of virulence factors such as proteases [124], lipases [125], enterotoxins [126] and hemolysins [127] by *Bacillus cereus* cause endophthalmitis, retinal layer folding and detachment within 12 h [10], with complete central visual loss, or entire eye loss often occur within 2 days [16]. The antibacterial widely used to combat the infections are amikacin [45], ceftazidime [96] and vancomycin [45,96] via intravitreal treatment. However, the aminoglycosides such as amikacin commonly used to combat sepsis serves as poor choice of antibacterial agent due to dose-dependent toxicity which may lead to destructive retinal microvasculitis [128]. In addition, the emergence of antimicrobial-resistant species, such as vancomycin-resistant *Enterococcus faecalis* and *Staphylococcus aureus* can transform the management of bacterial infections [48,53]. In addition, the broad-spectrum fluoroquinolones should not be utilized in intraocular therapy due to its potential toxicity [48,53]. Instead of subjecting the patients to the disadvantages posed by these prescribed antibiotics, stem cell therapy should be adopted to heal the infections more rapidly, thereby reducing the probability of gaining antimicrobial toxicity. 

Retinal infections may also be associated with syphilis caused by *Treponema pallidum*, which could infect individuals regardless of their immune status. Infected patients show manifestations such as retinitis, chorioretinitis and retinal vasculitis [97]. The flagellar filament outer layer protein (FlaA2) of *Treponema pallidum*, triggers the inflammatory reaction in monocytes by stimulating the signaling pathways involving toll-like receptor 2 (TLR2), myeloid differentiation primary response 88 (MyD88), extracellular-signal-regulated kinase (ERK), p38 and nuclear factor (NF)-κB, resulting in the release of pro-inflammatory cytokines IL-6 and activation of TNF [129,130]. Common antifungal used to treat infections due to *Treponema pallidum* include penicillin, ceftriaxone and doxycycline, which required up to 3 weeks of administration [58,64,97,98]. Stem cell intervention should be involved to shorten the treatment period, thereby reducing the period of time on which the bacteria exert its pathogenic effect and induce damage on host cells.

Other than that, *Mycobacterium tuberculosis* could also infect humans irrespective of immune status and give rise to retinal vasculitis. Conventional therapy starts with 2 months of rifampin, isoniazid and pyrazinamide intervention with or without ethambutol and followed by rifampin and isoniazid therapy. The completion of the regime will take up to 9 months [65]. On the other hand, multidrug-resistant tuberculosis associated infections are treated by streptomycin, capreomycin and quinolones [99]. Nevertheless, a study by Garhyan et al. showed that the *Mycobacterium tuberculosis* can reside in dormancy in bone marrow-mesenchymal stem cells (BM-MSCs) and persist in the intracellular milieu even after 3 months of extensive pyrazinamide and isoniazid treatment [131]. *Mycobacterium tuberculosis* can emerge and cause relapse in the patient after the discontinuation of antibiotic treatment, thus, rendering the therapy inadequate [131]. Therefore, a powerful regime is urgently required to supplement conventional antimicrobial therapy, which could be sought in stem cells.

**Figure 3:**
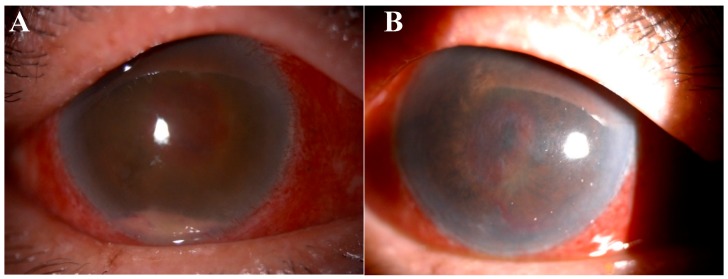
Right eye of patient. (**A**) On 2 May 2017, the patient demonstrated conjunctiva injection, hypopyon in the anterior chamber and yellowish material behind the lens. (**B**) On 30 May 2017, conjunctiva injection was still present after antimicrobial treatments and blood clot obscured the fundus view.

In another case reported in UKMMC, the patient complained of right eye blurring of vision without previous significant history of injury or trauma to the eyes. Clinical suspicion was of a bacterial infection. However, multiple samples from the vitreous was taken and revealed pus with no evidence of bacterial or fungal growth. Clinical examination revealed right eye circumcorneal injection, descement folds over the cornea and hypopyon in the anterior chamber (Figure 3A). Moreover, the fundus was obscured by a yellowish pus like material. Microbial insult to the delicate ocular tissue without the isolation of causative microorganisms is not surprising. In fact, it has been reported that patients infected with leptospirosis demonstrate ocular manifestation of uveitis which can manifest in either septic or aseptic form [132].

Despite rigorous treatment to the patient including topical, intravitreal and intravenous antibiotic and antifungal (fortum, gentamicin, vancomycin, amphotericin B), the vision did not recover and the patient demonstrated no perception to light (Figure 3B). The right eye was then subjected to evisceration (removal of eyeball content). In the events of ocular tissue damage where no visible microbial is observed, antimicrobials are proven to be ineffective in healing the ocular tissue. We suggest that stem cells treatment be considered to repair the damaged tissues in such a patient.

Altogether, the traditional antimicrobial management proved to be far from being effective in fighting against microbes infecting the human ocular tissue. This validates the urgency in developing potent alternative for the management of ocular microbial diseases, with stem cells as an attractive remedy option.

## 3. Stem Cell Therapy

In the past decades, the development of therapy to treat microbial infections experience major advances through the improvement of pharmacological strategies and the advancement of retinal prosthesis. These managements present rigid evidence that the pathogenic agents in the infected eye could be removed by prolonged antimicrobial treatments [64,65,66], or by surgical methods [25]. However, the loss of visual function caused by photoreceptor or RPE damage failed to be restored due to the lack of stem cells or tissue progenitors [133]. The limitations of present strategies validate the necessity of the innovation of an advanced healing method in stem cells, which have emerged as a crucial component of antimicrobial and regenerative medicine remedies. While the relatively easy accessibility of the retina renders it vulnerable to microorganisms’ attacks, it also appears as an excellent candidate for stem cell therapy. 

Our group has previously done extensive studies and reviews on stem cells [134,135,136,137]. Stem cells are undifferentiated cells that have the inherent ability in giving rise to multiple cell phenotypes and can be categorized into embryonic stem cells and adult stem cells. Adult stem cells can be isolated from many adult tissues, such as bone marrow and adipose tissue. They are generally acknowledged to have retained the capacity to differentiate into functional cells of mesodermal lineage, including neural and retina cells [30,31,32]. Over the past decades, the beneficial effects of stem cells are believed to reach beyond its regenerative potential and have found to be useful in fighting an array of septic infections.

### 3.1. Direct Microbial Clearance by Stem Cells

Mesenchymal stem cells (MSCs) exhibit direct antimicrobial properties which is mediated by the secretion of antimicrobial peptide LL-37 or also known as human cathelicidin antimicrobial peptide-18 (hCAP18) to combat the invading microorganisms [138,139,140]. The effector molecule LL-37 was reported to be a systemic control against viruses [141], fungus [142], Gram-negative (*Escherichia coli* and *Pseudomonas aeruginosa*) and Gram-positive (*Staphylococcus aureus*) bacteria [141,143,144,145,146,147,148,149]. Clinical results showed LL-37 mediated antimicrobial activity in cerebrospinal fluid of patients with infectious meningitis [150]. Besides displaying bactericidal activity through disintegration of microorganism cell membranes, LL-37 is also able to down-regulate plasma levels of endotoxin and cytokines [143]. The control of MSCs against bacteria and parasites in human was also demonstrated by the upregulation of indoleamine 2,3-dioxygenase, an enzyme with the capability to regulate the activity of T-cells [151,152,153,154,155]. Recently, the use of MSCs in cystic fibrosis murine model [156] has successfully decreased weight loss, circulating immune cells and chronic infection. The direct influence on the recruitment of inflammatory cells and microbe control suggest stem cell therapy as a potentially promising approach in combating ocular microbial infections.

Significant progress has been made in treating infected animals by inhibiting the growth of bacteria. In vivo delivery of bone marrow-MSCs indicated decreased pathogenic colony-forming unit and proliferation of bacteria in bronchoalveolar lavage fluid and lung homogenates in acute lung injury murine [28]. An increase in systemic pro-inflammatory cytokines was noted concurrently with MSCs-mediated anti-inflammatory response in limiting lung injury. Acute inflammatory reaction evoked by endotoxin and neutrophil infiltration in the lung was attenuated as early as 6 h and not exceeding 48 h following stem cells treatment [157]. Sepsis-driven inflammation in mice models was limited by MSCs-mediated down-regulation of alveolar inflammatory cell infiltration and pro-inflammatory mediator levels. Lung injury and dysfunction was also avoided by inhibition of apoptosis and improved bacteria clearance in vivo, which was suggested to reduce sepsis-related morbidity and mortality [26,27,28]. Treatment by MSCs demonstrated protection from septic shock by blood monocyte-mediated improved phagocytosis [29]. Administration of human bone marrow- or adipose-derived MSCs was shown to mitigate inflammation and enhanced survival in diseases such as experimental arthritis [158,159], colitis [145,160] and autoimmune encephalomyelitis [161]. These studies indicate the feasibility and usefulness of stem cell therapy as therapeutic tool in combating sepsis by the efficient management of inflammation. Thus, these unique characteristics of stem cells in killing pathogens could be utilized as host defense in infections affecting the ocular tissue. 

There are several clinical trials on stem cell therapy against sepsis according to the United States National Institutes of Health trial database (Available online: www.clinicaltrials.gov), including Netherlands phase 1 study using human allogenic adipose-derived MSCs (NCT02328612; available online: http://clinicaltrials.gov/) and Ottawa Hospital Research Institute phase 1 trial utilizing allogeneic MSCs (NCT02421484; available online: http://clinicaltrials.gov/). A pilot study of MSCs treatment on organ failure due to septic shock has also been registered by Central Hospital, France (NCT02883803; available online: http://clinicaltrials.gov/). A phase 2 randomized clinical trial has been carrying out by Russian National Research Center for Hematology on MSCs therapy for organ dysfunction and 28-day mortality in patients with septic shock and severe neutropenia (NCT01849237; available online: http://clinicaltrials.gov/). Stem cells administration to heal CMV infection (NCT002083731; available online: http://clinicaltrials.gov/) and H7N9 virus infection-associated acute lung injury (NCT02095444; available online: http://clinicaltrials.gov/) have been conducted as phase 2 trials. In addition, several clinical trials are ongoing for the treatment of inflammatory bowel diseases including Crohn Disease using bone marrow-MSCs and hematopoietic stem cells (NCT01851343, NCT01915927, NCT02225795 and NCT01540292; available online: http://clinicaltrials.gov/). The clinical trials of stem cells management in the fight against sepsis and inflammatory diseases in other organs validate the feasibility of stem cells treatment against ocular microbial infections. The use of stem cell therapy in treating microbial infection in the eye is ought to be more efficient and required a shorter treatment period compared to the traditional antimicrobials therapy adopted currently.

### 3.2. Modulation of Tissue Remodeling by Stem Cells

Another mechanism by which stem cells exert protective and reparative effect is through the modulation of tissue remodeling by reducing fibrosis in ocular tissue after injury. This peculiar benefit of stem cells can guard the eyes against the irreversible opacity due to the inflammatory reactions towards insulting pathogens. Fibrosis is the over proliferation and scarring of tissues by the excessive extracellular matrix deposition due to persistent inflammation in injured tissue and the subsequent tissue healing [18]. Fibrotic diseases, post-cataract surgery fibrosis and corneal or conjunctiva scarring can cause opacification which in turn leads to vision loss in patients [11]. Stem cells can release paracrine factors, which possess antifibrotic properties, important for the protection of the ocular tissues from fibrotic attack and prevention of blindness. Injured tissue undergoes healing by the influx of growth factors, cytokines, chemokines [17] and the stimulation of neutrophils and monocytes to eradicate pathogens, infected cells and fibrin clot. Macrophages differentiation activate fibrogenesis and angiogenesis [19], which lead to re-epithelialization process and secretion of connective tissue proteins such as vimentin and collagens I and III. Progressive fibrotic response in the ocular tissue drastically blurs vision as the passage of light to the eyes is obstructed. 

Tissue fibrosis can be attenuated by the capability of matrix remodeling in stem cells, which promote proper scar-less repair. The MSCs anti-fibrotic properties are demonstrated by hepatocyte growth factor (HGF) secretion which reduces fibroblasts expression of transforming growth factor (TGF)-β1 [162], collagen type I [163] and type III [164], important for regulation of matrix remodeling during wound repair. HGF elevation of fibroblasts matrix metalloproteinases (MMPs) expression such as MMP-1, MMP-2, MMP-3 and MMP-13 [165], tissue inhibitors of matrix metalloproteinases (TIMP) including TIMP-1 and TIMP-2 and the matricellular proteins thrombospondin-1 and tenacin C [166]. Simultaneously, decreased MMP-2 and MMP-9 protein expression [167] can promote matrix remodeling to restore the wound to its original state of integrity. HGF also facilitates keratinocyte expansion [168] while enhancing the expression of vascular endothelial growth factor (VEGF)-A to stimulate MMPs activities [169]. In addition, IL-10 released by stem cells can reduce TGF-β1 expression of macrophages and T-cells [170], while inducing fibroblasts to up-regulate MMPs and down-regulate collagen expression [171]. Suppression of pro-inflammatory cytokines such as IL-6 and IL-8 [172] at injured site by IL-10 can inhibit the accumulation of excessive collagen [173]. Furthermore, stem cells manufacturing of HGF [174] and prostaglandin E_2_ (PGE_2_) [175] prevent endothelial cells adjacent to injured blood vessels to undergo epithelial-to-mesenchymal transition (EMT) to become wound repairing fibroblasts [176], thus inhibiting fibrogenic reaction.

Overall, MSCs production of growth factors (HGF) [177,178], cytokines (IL-1β, IL-13, IL-10, IL-21, TGF-β1) [179] and chemokines (monocyte chemotactic protein 1 (MCP-1), macrophage inflammatory protein (MIP)-1β) [162,180,181] prevent fibrotic and scar tissue formation. Stem cells capability in preventing and reducing fibrosis have been proven in numerous studies. Treatment with stem cells led to decreased liver fibrosis [182,183] in murine models and ameliorate fibrosis due to lung [184,185] and kidney injury [186,187,188,189,190]. Antifibrotic effects were also shown in treatment of myocardial infarction [191,192], cardiomyopathy [167,193], heart failure [194] and cardiomyocytes differentiation [195]. Thus, stem cells should be administered to patients with microbial infections to reduce fibrosis and opacification of the ocular tissue by its modulation effect during tissue remodeling.

### 3.3. Immunomodulatory Effects of Stem Cells

Other than antimicrobial properties, stem cells also exhibit potent immunomodulatory function which is beneficial in resisting infections. Prolonged inflammation has seen presence of CD4^+^ and CD8^+^ T-lymphocytes, is devastating and prone to result in severe ocular tissue injuries [1,5]. Immunomodulatory effects of stem cells are the cumulative action of many molecules in the abrogation of T-cells expansion in suppressing the adverse effect of inflammation on tissue damage [145,159,160,161,196,197,198,199]. MSC-driven immunoregulatory properties was indicated by the reduction in inflammatory cell counts, protein and MIP-2 levels in the bronchoalveolar lavage fluid and increased bacterial clearance in acute lung injury studies [27,144,200,201]. The mechanisms underlying the therapeutic effects of stem cells have been described, including stem cells expression of chemokines and receptors which have the homing capacities to damaged tissue sites [202,203]. MSCs activated by IFNγ proinflammatory cytokine, alone or together with TNFα, IL-1α or IL-1β also facilitate immunosuppressive activity [204,205].

Upon TNFα or IFNγ stimulation, MSCs secrete high levels of PGE_2_ to constrain T-cell mitogenesis and IL-2 release, while reprogrammed monocytes and macrophages to induce IL-10 secretion and T-helper (Th) type 2 lymphocyte action [144,145,159,206]. Production of PGE_2_ also inhibits the maturation of dendritic cells, thereby suppressing T-cells stimulation [199]. The generated IL-10 can hinder the migration of neutrophils to tissues which would cause oxidative damage, thus alleviating multi organ injury [144]. The release of IL-10 also promotes human leukocyte antigen (HLA)-G5 release, which has also been reported to derive CD4^+^, CD25^+^ or CD8^+^ regulatory T-cells (T_REG_ cells) production with functional properties [207] and inhibit T-cell proliferation and cytotoxic effects [145,158,160,161,196,197,198]. 

The intrinsic properties of MSCs to influence the immune system were also shown by the suspension of lymphocytes and neutrophils apoptosis via IL-6 downregulation of reactive oxygen species [208,209], where IL-6 production also inhibit dendritic cells differentiation and maturation of monocyte and CD34^+^ hematopoietic progenitor cells, indicated by the reduction in cell-surface expression of major histocompatibility complex (MHC) class II and co-stimulatory molecules [210,211]. Following IL-6 generation, it is also indicated significant decrease in production of IFNγ, IL-2 and TNFα and increase of IL-4 secretion [206,210,212,213,214]. During sepsis, humoral factors’ production of stem cells impairs B-cells expansion and maturation [215], while blocking IL-2- or IL-15-driven natural killer cell proliferation [216]. Stem cells also have been reported to suppress Th1 cell proliferation and associated autoimmune and inflammatory responses [141]. These stem cells-derived anti-inflammatory mechanisms are crucial in providing protection against infection-induced ocular tissue injury. At present, the use of conventional antimicrobial therapy alone is insufficient and requires the simultaneous administration of anti-inflammatory drugs. Otherwise, the severe inflammatory reaction triggered by pathogens can cause edema and aggravate the visual function [5]. In contrast, immunomodulation by stem cell therapy is promising in alleviating the ocular conditions of patients with microbial infections.

### 3.4. Tissue Replenishing Property of Stem Cells

Potentials of stem cells in mitigating injuries are not restricted to microbial clearance and immunomodulation but also through tissue replenishment in repairing damaged site. In the last decade, the field of retinal research has made significant advances, specifically in treating blindness due to retinal degenerative diseases. Embryonic stem cells [217] and bone marrow-derived MSCs [218] can be induced into retinal lineage. Recently, RPE was successfully differentiated from adipose tissue-derived MSCs [31,32] useful for replenishing the degenerated or trauma-injured RPE. Sub retinal transplantation of photoreceptors differentiated from MSCs has also been demonstrated in retinal degeneration rat model [219]. Furthermore, stem cells are able to protect against photoreceptor degeneration by secreting neurotrophic factors [220] which promote tissue repair and regeneration, through fine regulation of mitogenic, angiogenic, anti-apoptotic and scar reduction activities. 

Even though traditional antimicrobials therapy could eliminate the microbes in the eyes, ocular tissue that was damaged during infection could never be restored and pose permanent visual disturbance in patient [22,23]. Stem cell therapy is promising in regenerating damaged ocular layers and therefore should be focused as the optimum treatment option in the future. In addition, the powerful immunosuppressive nature of stem cells permits autologous and allogeneic transplantation in the absence of pharmacological immunosuppression or genetic modification by the inhibition of pro-inflammatory cytokines production [221]. The concern of ethical issues [222] and teratoma formation are also obsolete [223] specifically in the application of MSCs. In light of these advantages, the potential uses of stem cells as therapeutic tool in curing ocular microbial infections is highly appealing. 

In principle, vision can be restored in microbial-infected damaged eyes. This serves as an attractive and novel management as the replacement of photoreceptor cells and RPE will be able to restore the native visual-processing pathways for normal visual perception. Stem cells as the perfect cell source offer significant potential to expand and differentiate into functional and viable photoreceptor cells and RPE in microbial infections damaged ocular tissue. The addition of stem cells to antimicrobials treatment is poised to have a higher efficacy in suppressing microbial growth, reducing retinal inflammatory response and accelerating tissue healing due to microbial infections compared to the conventional antimicrobials only treatment. This strategy directly addresses the persisting limitations of traditional treatments in facilitating the return of visual functions in affected individuals. Thus, we believe that significant advancements in ocular microbial infections management can be made by implanting stem cells into the ocular tissue together with the conventional antimicrobial treatment to reduce inflammation on the eye, repair ocular damages and ultimately restore eyesight of the patients. 

## 4. Conclusions

This article reviewed the list of pathogens commonly infecting the eye and the current antimicrobial strategies along with their therapeutic limitations. We also provided recent updates of stem cells advancements in resisting sepsis and suggested stem cell therapy as the powerful alternative regime for ocular microbial infections. In summary, microbial infections and the resulting inflammation of the eye are stoppable by conventional antimicrobial therapy, however, the damages exerted are not reversible. Stem cells approach to replace damaged ocular tissue by photoreceptor cells and RPE proliferation and differentiation offers tremendous potential for enhanced microbial-damaged ocular tissue repair. Ultimately, the antimicrobial function and regenerative properties of stem cells could be exploited to treat ocular damage caused by severe infection and restore vision in microbial-infected patients. 

## Figures and Tables

**Table 1:** The causative agents of ocular microbial infections, antimicrobial treatments, route and duration of administration.

Microbial Infections	Species	Infections	Antimicrobial Treatments	Route of Administration	Duration of Administration	Reference
Fungal Infections	*Candida albicans*	chorioretinitis	caspofungin, micafungin, or anidulafungin	Intravenous or oral	Approximate 1 month	[7]
	*Aspergillus fumigatus*	retinitis, invasive aspergillosis	voriconazole, or posaconazole	Intravenous or oral	-	[7,33,87,88]
	*Cryptococcus neoformans*	multifocal chorioretinitis	flucytosine and amphotericin B	Intravenous or oral	-	[84,85]
	*Histoplasma capsulatum*	histoplasmosis, retinitis	Laser cauterization	-	Repeated	[2,64]
Viral Infections	CMV	retinitis	ganciclovir	Intravenous, intravitreous	>3 weeks	[21]
			foscarnet	Intravenous	-	[57]
			cidofovir	Intravenous	-	[62]
			fomivirsen	Intravenous	-	[89]
	VZV, HZV, HSV types 1 and 2	ARN, PORN	acyclovir	Intravenous	7–12 weeks	[62,67]
			foscarnet	Intravitreal	-	[20]
			valaciclovir	Oral	-	[62,90]
			famciclovir	Oral	-	[62,91]
Parasitic Infections	*Toxocara canis*, *Toxocara cati*	ocular toxocariasis	albendazole or thiabendazole	-	-	[37,38,92]
	*Toxoplasma gondii*	ocular toxoplasmosis	pyrimethamine-sulfadiazine, trimethoprim-sulfamethoxazole or pyrimethamine-azithromycin	-	-	[93,94,95]
Bacterial Infections	*Enterococci*, *Streptococci*, *Bacilli*, gram-negative bacteria	retinitis	vancomycin-amikacin or vancomycin-ceftazidime	-	-	[45,96]
	*Treponema pallidum*	ocular syphilis	penicillin	Intravenous	14 days	[58,97,98]
			ceftriaxone or doxycycline	Parenteral	3 weeks	[66,98]
	*Mycobacterium tuberculosis*	tubercular retinal vasculitis	isoniazid, rifampin and pyrazinamide, with or without ethambutol	-	Up to 9 months	[65]
			streptomycin, capreomycin, or quinolones	-	-	[99]
